# BCL2A1 regulates Canady Helios Cold Plasma-induced cell death in triple-negative breast cancer

**DOI:** 10.1038/s41598-022-07027-4

**Published:** 2022-03-08

**Authors:** Saravana R. K. Murthy, Xiaoqian Cheng, Taisen Zhuang, Lawan Ly, Olivia Jones, Giacomo Basadonna, Michael Keidar, Jerome Canady

**Affiliations:** 1 Jerome Canady Research Institute for Advanced Biological and Technological Sciences, Takoma Park, MD USA; 2 Plasma Medicine Life Sciences, Takoma Park, MD USA; 3 University of Massachusetts School of Medicine, Worcester, MA USA; 4 The George Washington University, Washington, DC USA; 5 Holy Cross Hospital, Department of Surgery, Silver Spring, MD USA

**Keywords:** Biochemistry, Biophysics, Cancer, Cell biology, Molecular biology, Oncology

## Abstract

Breast cancer is the leading cause of cancer death among women. Triple-negative breast cancer (TNBC) has a poor prognosis and frequently relapses early compared with other subtypes. The Cold Atmospheric Plasma (CAP) is a promising therapy for prognostically poor breast cancer such as TNBC. The Canady Helios Cold Plasma (CHCP) induces cell death in the TNBC cell line without thermal damage, however, the mechanism of cell death by CAP treatment is ambiguous and the mechanism of resistance to cell death in some subset of cells has not been addressed. We investigate the expression profile of 48 apoptotic and 35 oxidative gene markers after CHCP treatment in six different types of breast cancer cell lines including luminal A (ER^+^ PR^+/−^HER2^−^), luminal B (ER^+^PR^+/−^HER2^+^), (ER^−^PR^−^HER2^+^), basal-like: ER^−^PR^−^HER2^−^ cells were tested with CHCP at different power settings and at 4 different incubation time. The expression levels of the gene markers were determined at 4 different intervals after the treatment. The protein expression of BCL2A1 was only induced after CHCP treatment in TNBC cell lines (p < 0.01), whereas the HER2-positive and ER, PR positive cell lines showed little or no expression of BCL2A1. The BCL2A1 and TNF-alpha expression levels showed a significant correlation within TNBC cell lines (p < 0.01). Silencing BCL2A1 mRNA by siRNA increased the potency of the CHCP treatment. A Combination of CHCP and CPI203, a BET bromodomain inhibitor, and a BCL2A1 antagonist increased the CHCP-induced cell death (p < 0.05). Our results revealed that BCL2A1 is a key gene for resistance during CHCP induced cell death. This resistance in TNBCs could be reversed with a combination of siRNA or BCL2A1 antagonist-CHCP therapy.

## Introduction

Breast cancer is the most common cause of cancer death among women worldwide^1^ and it exhibits diverse molecular features that reflect the high heterogeneity which complicates the clinical treatment^2^. Breast cancers are categorized by the molecular receptor status^3^ that are expressed such as the estrogen receptor (ER), progesterone receptor (PR), and human epidermal growth factor receptor 2 (HER2) expression. The prognosis for breast cancer patients is generally favorable with ER^+^/PR^+^ tumors, intermediate with either ER^+^/PR^−^ or ER^−^/PR^+^ tumors, but a devastating outcome for triple negative breast cancer (TNBC, ER^−^/PR^−^/HER2^−^). Based on the receptor status selective therapeutic interventions are carried out. For example, in the case of ER^+^ tumor estrogen-receptor modulators, such as tamoxifen and letrozole are administered^4^, trastuzumab (Herceptin) is a humanized monoclonal antibody developed to target and inhibit the function of HER2 and a dual anti-HER2 regimen, pertuzumab in combination with trastuzumab and docetaxel are administered, however, the incidences of adverse events and resistance to these drugs are not uncommon^5^. Triple negative breast cancer (TNBC) (ER^−^/PR^−^, HER2^−^) do not respond to endocrine therapy or for HER2-targeted therapies^6,7^. Therapies targeting TRAIL (TNF (tumor necrosis factor)-related apoptosis-inducing ligand)^8^ and cyclin-dependent kinases (CDK) or cell cycle regulators^9^ have been used with limited success. In recent years Cold atmospheric plasma (CAP) technology that utilizes ionized gas to selectively induce apoptosis in cancer cells has shown very encouraging results^10–13^. Preclinical In vivo studies in mouse models for various cancers, CAP treatment have demonstrated to effectively reduce tumor growth rate and induce cancer cell death. US Medical Innovations, LLC (USMI) and Jerome Canady Research Institute for Advanced Biological and Technological Sciences (JCRI-ABTS)™, LLC have developed Canady Helios Cold Plasma™ (CHCP) system and have received the Food Drug Administration (FDA) approval for the first clinical trial use for the treatment of cancer in the United States^11^. CHCP treatment induces apoptosis in various breast cancer cells and the potency of the treatment depends on a combination of parameters such as the concentration and the time of the plasma treatment^14^. Susceptibility or resistance to CHCP treatment is also determined by the molecular features of the cell types such as the receptor status which are classified into intrinsic subtypes including luminal A (ER^+^PR^+/−^HER2^−^), luminal B (ER^+^PR^+/−^HER2^+^), basal-like (ER^−^PR^−^HER2^−^), and HER2-positive (ER^−^PR^−^HER2^+^)^10,12^. It is perspicacious to understand downstream genes that induce susceptibility or resistance to CHCP treatment in these cells. At high concentrations and duration of CHCP treatment, most of the breast cancer cells often undergo apoptosis due to the release of reactive oxygen and nitrogen species (RONS) and oxidative stress-induced cell toxicity of these species^15^. The mechanism of such oxidative stress-induced cell death process is broadly discussed in various studies^16–18^. Our previous studies of CHCP treatment on subtypes of breast cancer cell lines showed that its ability to reduce breast cancer viability by 92–99% regardless of the status of the receptors on these cells at the most optimal power setting and time of treatment^12^. Optimizing CHCP treatment to lower power setting or shorter time would yield many practical clinical benefits. However, at a lower power settings or shorter time of the CHCP insult, some subset of cells resists cell death and survive, but the molecular mechanism for such survival in these cells has not been systematically investigated. An insight into the gene expression of the molecular marker for apoptosis and oxidative stress would significantly increase our understanding of cancer cell viability and the oncogenic processes that influence the response to CHCP therapy. This study was undertaken to identify genes that could predict response to CHCP treatment on subtypes of breast cancer cell lines and the genes that instigate resistance to cell death induced by CHCP treatment.

## Results

**Figure 1:**
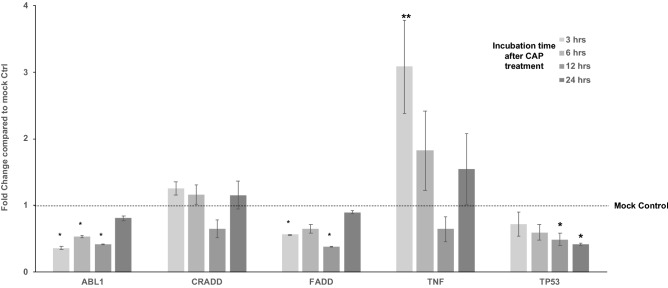
CHCP treatment differentially regulates the apoptosis induction genes. Bar graph showing the differential gene expression of genes involved in the induction of apoptosis after CHCP treatment in TNBC cell line MDA-MB-231. The samples were normalized to 18s rRNA and the fold changes were compared to mock controls. Error bars represent means ± SEM. Student *t* test significant at *p values < 0.05, **p values < 0.01.

**Figure 2:**
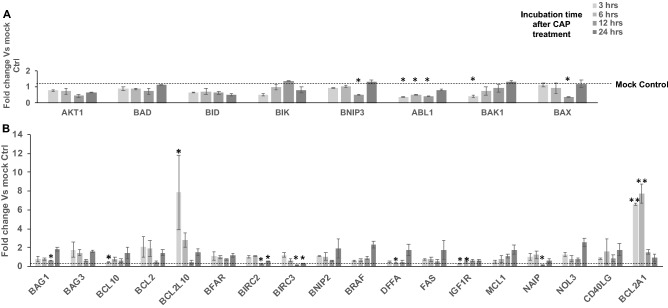
CHCP treatment differentially regulates the apoptosis regulation genes. Bar graph showing the differential gene expression of genes involved in the positive (**A**) and negative (**B**) regulation of apoptosis after CAP treatment in TNBC cell line MDA-MB-231. The samples were normalized to 18s rRNA and the fold change were compared to mock controls. Error bars represent means ± SEM. Student *t* test significant at *p values < 0.05, **p values < 0.01.

**Figure 3:**
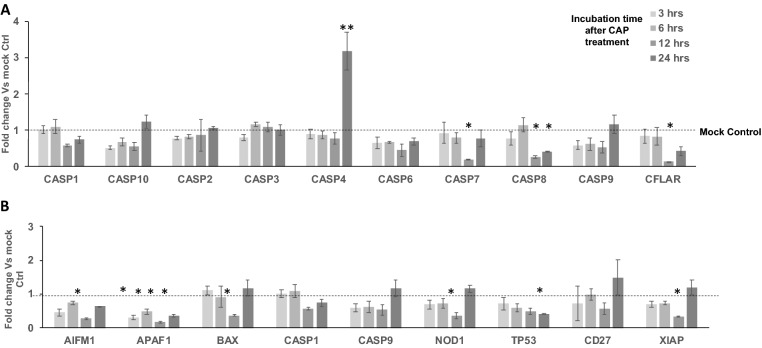
CHCP treatment differentially regulates Caspase genes. Bar graph showing the differential gene expression of genes involved in the caspase family (**A**) and caspase activation (**B**) regulation of apoptosis after CAP treatment in TNBC cell line MDA-MB-231. The samples were normalized to 18s rRNA and the fold changes were compared to mock controls. Error bars represent means ± SEM. Student *t* test significant at *p values < 0.05, *p values < 0.01.

**Figure 4:**
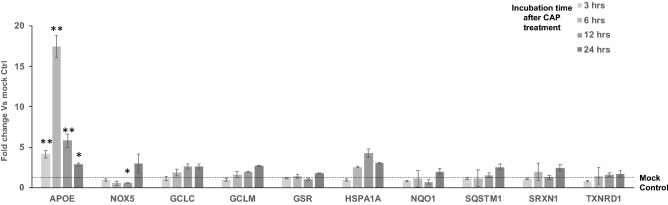
The regulation of oxidative stress genes APOE was differentially expressed after CHCP treatment. Bar graph showing the differential gene expression of genes involved in the regulation of oxidative stress after CAP treatment in TNBC cell line MDA-MB-231. The samples were normalized to 18s rRNA and the fold changes were compared to mock controls. Error bars represent means ± SEM. Student *t* test significant at *p values < 0.05, *p values < 0.01.

To systematically investigate the molecular basis for survival after CAP treatment by breast cancer cell lines which are classified into their intrinsic subtypes such as luminal A (ER^+^PR^+/−^HER2^−^), luminal B (ER^+^PR^+/-^HER2^+^), basal-like (ER^−^PR^−^HER2^−^), and HER2-positive (ER^−^PR^−^HER2^+^), we conducted quantitative real time PCR analysis to screen the genes that are differentially expressed after CHCP treatment. We selected genes from four major categories based on their ability in “induction of apoptosis”, “regulation of apoptosis”, “caspases & regulators” and “responders of oxidative stress” (Supplementary Table 1). For the purpose of screening, we CHCP treated TNBC cell line MDA-MB-231 cells at 80 and 120 powers for 5 min and incubated until total RNA was isolated at 3-, 6-, 12- and 24-h time points. Complete cell death was achieved at the optimal condition with CHCP treatment after 24 h of incubation, hence all the experiments were monitored until this time point. After total RNA isolation and first strand synthesis, qRT-PCR was carried out to analyze the mRNA expression of genes involved in the induction of apoptosis (Fig. 1), regulation of apoptosis (Fig. 2), caspase activation (Fig. 3) and regulation of oxidative stress (Fig. 4).

### CHCP treatment differentially regulates the induction of apoptosis genes

ABL proto-oncogene 1, non-receptor tyrosine kinase (ABL1), Fas-associated via death domain (FADD) and Tumor Protein p53 (TP53) mRNA expression were significantly (p < 0.05) down-regulated after CHCP treatment compared to untreated mock samples, whereas death associated protein kinase 1 (DAPK1) expression was not detected in either of the sample groups. Tumor Necrosis Factor (TNF) alpha was significantly upregulated after 3 h of treatment (p < 0.01). Although not statistically significant, 6 h and 24 h sample groups showed an upward trend in expression, suggesting that TNF-alpha is involved in the induction of apoptosis in CHCP treated breast cancer cells (Fig. 1).

### CHCP treatment differentially regulates the apoptosis regulation genes

Among genes involved in apoptosis regulation, B-cell lymphoma 2-related protein A1 (BCL2A1)/Bcl-2-related gene expressed in fetal liver (Bfl-1) and BCL2 like 10 (BCL2L10) mRNA transcripts showed significant up-regulation (p < 0.001 and p < 0.05) (Fig. 2B). BCL2A1 mRNA expression above background noise was observed only after CHCP treatment and its expression was at least 4 folds higher at 3- and 6-h incubation time points after the treatment. AKT serine/threonine kinase 1 (AKT1) mRNA expression was downregulated throughout all the incubation time points after treatment (p < 0.05). BCL2 associated agonist of cell death (BAD) mRNA expression was relatively unchanged. Whereas BH3 interacting domain death agonist (BID) mRNA expression was downregulated throughout the 24 h time point (p < 0.05). BCL-2 Interaction Killer (BIK) mRNA expression was downregulated at 3 h time point (p < 0.05) but steadily recovered above the baseline expression in 12 h and 24 h sample groups. BCL2 interacting protein 3 (BNIP3) mRNA expression was relatively unchanged at 3 and 6 h sample groups but was down regulated at 12 h and recovered above the baseline expression at the 24 h time point. BCL2 associated X, apoptosis regulator (BAX) mRNA expression remained at baseline levels at 3-, 6- and 24-h incubation time point but was significantly down regulated at 12 h (p < 0.05). The differential mRNA expressions of B-cell lymphoma 2 (BCL2), BCL2 associated athanogene 1 (BAG1) and BCL2 like 10 (BCL2L10), Bifunctional apoptosis regulator (BFAR), BCL2 family apoptosis regulator (MCL1), Nucleolar protein 3 (NOL3), and BCL2 associated athanogene 3 (BAG3) were not statistically significant in response to the CAP treatment (Fig. 3B).

### CHCP treatment differentially regulates caspases genes

Except for CASP4 all the other caspases that we analyzed were either unchanged or down regulated after CAP treatment (Fig. 3A). CASP4 mRNA was up-regulated at least ~ 4 folds at 24 h incubation time point after CHCP treatment (p < 0.01).

### CHCP treatment differentially regulates the oxidative stress genes

Among all the genes involved in oxidative stress (NOX5, GCLC, GCLM, GSR, NQO1, SQSTM1 and TXNRD1), Apolipoprotein E (APOE) was the only genes which was up-regulated significantly (p < 0.001) after CAP treatment at 3-, 6-, 12-, and 24-h incubation time points.

### BCL2A1 gene up-regulation after CHCP treatment

**Figure 5:**
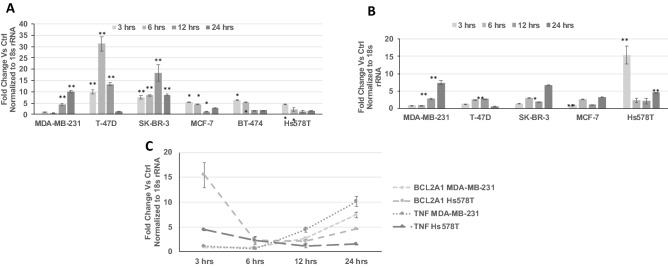
Differential expression TNF alpha and BCL2A1 are correlated after CHCP treatment. Line graph showing the differential gene expression of TNF (**A**) and BCL2A1 (**B**) transcripts after CAP treatment at 120 power for 5 min on TNBC cell line MDA-MB-231. The samples were normalized to 18s rRNA and the fold changes were compared to mock controls. Line graph (**C**) shows the correlation of BCL2A1 and TNF gene expression after CAP treatment at 120 power for 5 min on two TNBC cell line MDA-MB-231 and Hs578T cells. The samples were normalized to 18s rRNA and the fold changes were compared to mock controls. Error bars represent means ± SEM. Student *t* test significant at *p values < 0.05, *p values < 0.01.

Based on the initial gene profile screening for the CAP treated TNBC cells (MDA-MB-231 cells), three genes were selected and further analyzed in other breast cancer cell lines MCF-7 (ER^+^, PR^+^, HER2^−^), T-47D 7 (ER^+^, PR^+^, HER2^−^), SK-BR-3 (ER^−^, PR^−^, HER2^+^), BT-474 (ER^+^, PR^+^, HER2^+^), MDA-MB-231 (ER^−^, PR^−^, HER2^−^) and Hs574T (ER^−^, PR^−^, HER2^−^) (Fig. 5) were carried out. Hs574T a TNBC cell line, showed significant up regulation of BCL2A1 at 3 h after CAP treatment and remained above the twofold level for up to 24 h. T-47D 7, SK-BR-3, and BT-474 had very low detection levels of BCL2A1 expression. MCF-7 showed a significant twofold up regulation of BCL2A1 at 6- and 24-h time points. TNF was significantly up-regulated in all the cell lines at different incubation time points after CAP treatment. The trend of BCL2A1 and TNF expressions were correlated in MDA-MB-231 and Hs574T cells suggesting a synergy in their regulation (Fig. 5C).

**Figure 6:**
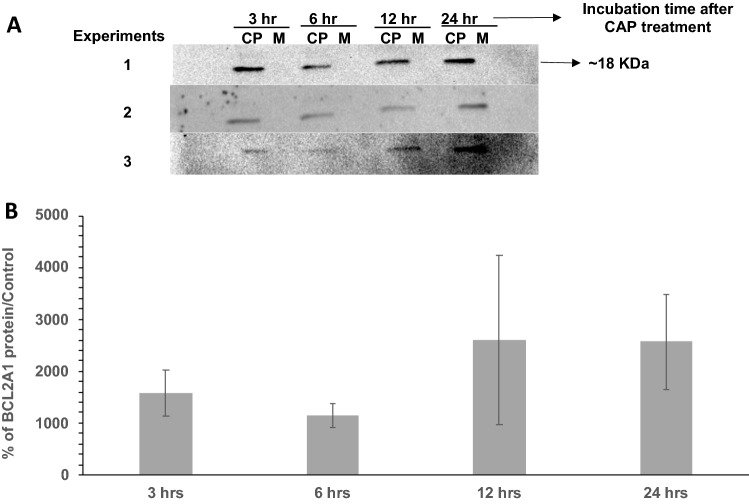
BCL2A1 protein if only expressed after CHCP treatment in TNBC cells. Expression of BCL2A1 after CAP treatment (CP) and mock controls (M) in TNBC cell line MDA-MB-231. Representative Western blots (**A**) and bar graph representation (**B**) of the quantification of BCL2A1 protein in total tissue lysates from MDA-MB-231 cells after CHCP treatment compared to the mock control groups.

Western blot analysis also revealed that BCL2A1 protein was expressed only after CHCP treatment in TNBC (MDA-MB-23) cells and the protein expression was detectable throughout the 24-h incubation period whereas mock control cells did not show any detectable protein bands (Fig. 6), suggesting that BCL2A1 expression occurs only after induction of cellular stress induced by CHCP treatment. However, there was no significance in protein expression between 3-, 6-, 12- and 24- hour sample groups after CAP treatment.

### Silencing of BCL2A1 expression increases CHCP treatment potency

**Figure 7:**
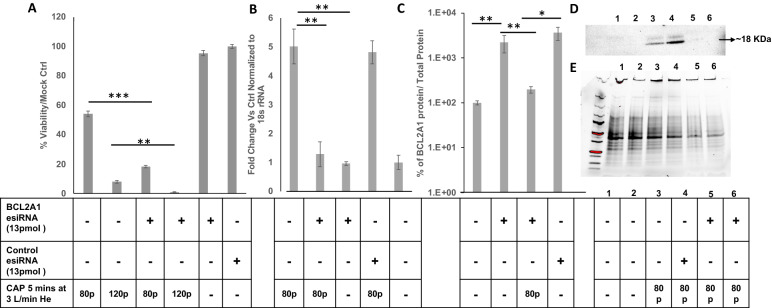
Silencing of BCL2A1 mRNA increases CHCP treatment potency. Bar graphs showing the (**A**) effects of BCL2A1 esiRNA silencing on cell viability and (**B**) the fold change of BCL2A1 mRNA and (**C**) protein expression in MDA-MB-231 cell line in combination with CAP treatment. At 24 h. after transfection with BCL2A1 esiRNA (13 pmol), as mentioned in the methods section survival of treatments was determined by MTT assay and BCL2A1 fold change by qRTPCR and western blots. (**D**) and (**E**) show a representative blot for BCLA1 and total protein respectively. The error bars represent means ± SEM. (n = 3). (****p < 0.0001, *p < 0.05, **p < 0.01, ***p < 0.001, Student’s *t*-test) versus control.

Specific downregulation of BCL2A1 mRNA and protein expression by esiRNAs silencing in MDA-MB-231 cells were carried out to determine the effects of BCL2A1 with and without CHCP treatment and were analyzed by qRTPCR (Fig. 7B) and western blot (Fig. 7C–E). Approximately a ~ fivefold decrease in BCL2A1 at mRNA expression level and ~ 95% knock out at protein levels were observed after esiRNA-BCL2A1 compared to control esiRNA treatment. The cell viability for all the sample groups was determined by MTT assay (Fig. 7A), esiRNA-BCL2A1 had no effect on the MDA-MB-231 cells, but in combination with the CHCP treatment at 80p 5 min the viability of cells significantly (p < 0.001) reduced after 24 h compared to either of the treatments alone (Fig. 7A). The CHCP treatment setting at 80p 5 min was chosen based on our previous studies^12^.

### Silencing of BCL2A1 expression increases cell death at G1 phase of the cell cycle after CHCP treatment

**Figure 8:**
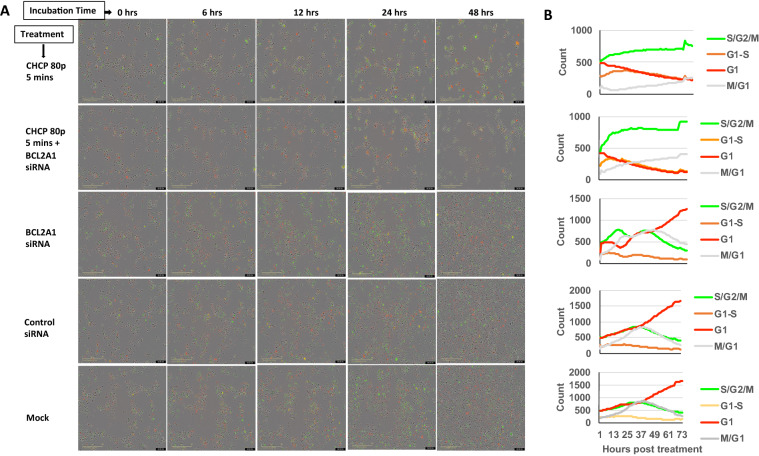
Silencing of BCL2A1 expression increases cell death at G1 phase of the cell cycle after CHCP treatment. (**A**) Representative pictures of IncuCyte live cell image showing the live state of MDA-MB-231-Cell Cycle Green/Red reagent stable cells treated with/without CHCP treatment or siRNA BCL2A1. The CHCP treated cells recover 50% of the time but when transfected with siRNA-BCL2A1 in combination with CHCP, such recovery is inhibited. (**B**) Shows the line graph for the quantification of the cells in G1, G1-S, S/G2/M, and M-G1 phases after CHCP treatment corresponding to section A.

For live cell image analysis, stables of MDA-MB-231 cells expressing Cell Cycle Green/Red tags were created. The Incucyte Cell Cycle Green/Red tag is a fluorescent, single cassette indicator expressing both the GFP (green fluorescent protein) and mKate2 (red fluorescent protein) to distinguish between cells in the G1 and S/G2/M cell cycle phase without altering cell function. The Incucyte live cell imaging experimental setup is detailed in the method section. MDA-MD-231 stable cells were monitored for cell confluence and G1 (Red) or S/G2/M (Green) or S to G1 transition (S-G1, Yellow) phases of the cell cycle for up to 3 days by Incucyte live imaging. The cells treated with esiRNA-BCL2A1 did not show any changes in cell confluence or cell cycle phases compared to mock cells or esiRNA-control treated cells, but when treated with CHCP at 80p 5 min the viability of esiRNA-BCL2A1 treated cells were close to 0% by 12 h and remained the same throughout the incubation period, whereas in CHCP only treated group showed 30% viability at 12 h and recovered to 50% and 70% viability by 24 and 48 h incubation time points respectively (Fig. 8). The reduction in a number of cells at the G1 phase was very pronounced in stable MDA-MB-231 cells with esiRNA-BCL2A1-CHCP treatment compared to CHCP treatment alone. The number of cells transitioning from G1 phase to S/G2/M phases was close to 0% in esiRNA-BCL2A1-CHCP treated cells whereas the CHCP treatment alone group showed a limited number of cells progressed towards S/G2/M phases (yellow and green) compared to the control groups (Fig. 8B). After CHCP treatment at 80p 5 min, the MDA-MB-231 cells at the G1 phase were drastically reduced compared to control groups. There was an increase in cells at the G1-S phase during initial time points but later these numbers were reduced. The cells at S/G/M phase increased and reached a plateau stage by 12 h. All these changes were more pronounced in cells treated with siRNA-BCL2A1 in combination with the CHCP treatment. In the siRNA-BCL2A1-CHCP treated group, ~ 99% of the cells were dead by 24 h and the cell death occurred when the cells reached S/G/M phase. Even though there was no detectable BCL2A1 protein at the baseline level in MDA-MB-231 cells, siRNA-BCL2A1 without the CHCP treatment also showed a small increase in the S/G/M phase cells compared to siRNA control and mock groups.

### Combination of the BET bromodomain inhibitor CPI203 and CHCP treatment increases TNBC cell death

**Figure 9:**
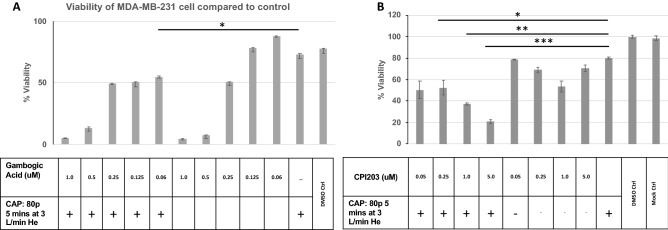
Combination of the BET bromodomain inhibitor CPI203 and CHCP treatment increases TNBC cell death. Viability of TNBC cell line MDA-MB-231 with Gambogic acid, an antagonist of antiapoptotic Bcl-2 family (**A**) or CPI203, a BET bromodomain inhibitor after CAP treatment. Bar graph represents MDA-MB-231 MTT cell viability assay after treatment with Gambogic acid (**A**) or CPI203 in the combination of with/without CAP treatment at 80p 5 min at 3 L/min He. The samples were compared to the mock control groups. The data is represented by the SEM (n = 3). (*p < 0.05, **p < 0.01, ***p < 0.001, Student’s t-test).

To determine the effects of BCL2A1 antagonist drugs in combination with the CHCP treatment on TNBC cell death, gambogic acid (GA), an antagonist of antiapoptotic Bcl-2 family, and CPI203, a BET bromodomain inhibitor were tested. CPI203 has previously been shown to downregulate BCL2A1 expression in a small-molecule antagonist of BCL-2 (ABT-199) drug-resistant diffuse large B-cell lymphoma (DLBCL)^19^. Baseline toxicity measurements were established for GA and CPI203 on MDA-MB-231 cells. Cell viability with GA (5 dose concentrations) and CPI203 (4 dose concentrations) in combination with CHCP treatment on MDA-MB-231 cells were tested and measured by MTT assay. GA showed decreased cell viability in all the 5 doses tested but none of these doses significantly reduced the cell viability in combination with CAP treatment compared to CAP treatment or GA alone (Fig. 9). CPI203 showed significant reduction in cell viability at three dose concentrations (0.25 µM; *p < 0.05), (1 µM; **p < 0.01) and (5 µM; ***p < 0.001) compared to CAP treatment or the CPI203 alone and the increase in the cell death corresponded to the increase in the CPI203 concentrations (Fig. 9).

## Discussion

CHCP, a promising anti-cancer treatment has been demonstrated in several cancers including TNBC^12,14^. Previous studies have shown that molecular features and corresponding tumor subtypes of each breast tumor cell lines are feasible as models for tumors of the same subtypes^20^. In this study, we are the first to demonstrate gene expression profile screening for 6 different breast cancer cell lines with different molecular subtypes and show that BCL2A1 was only expressed after CHCP treatment in TNBC and silencing of BCL2A1 by siRNA or inhibit its expression by CPI203 increased the potency of CHCP treatment. This study also shows that narrowing down and analyzing a few categories of genes would be very helpful to pinpoint low transcript number expression genes that would express only in certain conditions such as CHCP treatment. Here, by analyzing four different categories of genes including induction of apoptosis, regulation of apoptosis, caspases and regulators, and reactive oxygen species (ROS), and metabolism specific expression of low transcript number gene such as BCL2A1 gene expression were detected after CHCP treatment. This would have not been possible if a high throughput gene expression study were carried out. In fact, Western blot analysis revealed that detectable BCL2A1 protein was only expressed after CHCP treatment (Fig. 6).

BCL2A1 protein contains 4 BH-domains and does not have any C-terminal transmembrane domain like other BCL2 family of protein but regulates anti-apoptotic function^21^. BCL2A1 mRNA expression has been shown to be generally overexpressed in many cancers including breast cancer^22,23^ and is attributed to poor prognosis^24^ and directly contributes to chemoresistance^25^. Studies have shown that CD40, PI3K and ERK signaling initiated by ICAM-1 binding has been found to induce NF-kB and subsequently BCL2A1 expression^26^. With preliminary experimental results, we did not see any increase in NF-kB (preliminary results not shown). Nutlin-3, a cis-imidazoline analog, and p53 dependent protein has been shown to regulate the expression on BCL2A1^27^, but in our studies p53 was downregulated after CHCP treatment, hence no further analysis of nutlin protein was carried out. Apart from these factors, BCL2A1 has been shown to be regulated by other transcription factors such as all-trans retinoic acids^28^, WT1, or PU1^23^. However, we found a very close correlation between the TNF expression and BCL2A1 expression in two TNBC cell lines (Fig. 5C).

TNF-alpha is a cell signaling protein (cytokine) involved in apoptotic cell death by extrinsic apoptotic signaling pathway via death domain receptors and in the promotion and progression of cancer, including TNBC cells^29^. Previous studies have shown that TNF super family members are induced after CAP treatment in cancer cells^30^. In our study, TNF-alpha mRNA expression was significantly upregulated after CHCP treatment suggesting TNF is involved in both induction of apoptosis and in the survival of breast cancer cells after CHCP treatment (Fig. 1). In the case of T-47D 7 (ER^+^, PR^+^, HER2^−^) and SK-BR-3 (ER^−^, PR^−^, HER2^+^) which are the least resistant cell lines among all the cell lines treated with CHCP, the expression of TNF in these cell lines were up regulated after CHCP treatment but had very little detectable BCL2A1 mRNA. However, MDA-MB-231 (ER^−^, PR^−^, HER2^−^) and Hs574T (ER^−^, PR^−^, HER2^−^) TNBC cell lines which are comparatively more resistant to CHCP treatment had a correlative TNF-alpha and BCL2A1 mRNA expression after treatment. Previous studies have shown upregulation of BCL2A1 protein prevents apoptosis induced by various stimuli including TNF-alpha, TRAIL, Fas, and chemotherapeutic agents by directly binding to tBid and BAK^31–33^. Several studies have shown that an increase in TNF-alpha expression induces cell survival and cancer progression^34,35^. In fibrosarcoma cells for example, BCL2A1 was rapidly induced after TNF-α stimulation^25^. Studies have also shown that TNF-alpha gene knockout in the TNBC cell line induces apoptosis^36^. In our study, silencing of BCL2A1 by siRNA showed increased cell death within 12 h of CHCP treatment compared to CHCP treatment alone. The upregulation of BCL2A1 at the lower power setting of CHCP treatment increases the survival of the cells. The specific reactive oxygen and nitrogen species (RONS) that are generated during CHCP treatment that induces TNF-alpha expression in turn regulating BCL2A1 expression in the above breast cancer cells is a detailed stand-alone study by itself.

Furthermore, a drastic reduction in G1 phase cells and an increase in S/G2 phase cells compared to control groups after CHCP treatment at 80p 5 min is because of the histone mRNA degradation and a pause in cell division which in turn accumulates more cells at late S phase^37^. This phenomenon is more pronounced in cells treated with siRNA-BCL2A1 in combination with the CHCP treatment suggesting that silencing BCL2A1 expression increases cell death. In the siRNA-BCL2A1-CHCP treated group, almost all the cells undergo cell death by 24 h at the late S phase and there is a plateau phase at this stage because the fluorescent marker proteins were oxidized, and the fluorescence were retained with incomplete protein degradation. Moreover, even though there is no detectable BCL2A1 protein at the baseline level in MDA-MB-231 cells, siRNA-BCL2A1 without the CHCP treatment also showed a small increase in the cells at S/G/M phase compared to siRNA control and mock groups, suggesting that there is some undetectable functioning BCL2A1 protein at the baseline levels.

CPI203, a BET bromodomain inhibitor that was previously shown to downregulate BCL2A1^19^, increased cell death in TNBC when treated in combination with CHCP treatment compared to treatment alone. BCL2A1 belong to the anti-apoptotic subgroup of the BCL-2 family of proteins, and they bind to the BH3-only protein subgroup of this family (BIM, cleaved BID, PUMA, NOXA) bind to the pro-survival proteins, leading to the release of BAX and BAK from their anti-apoptotic counterparts. These events prevent the permeabilization of the mitochondrial outer membrane and the subsequent cytosolic release of cytochrome c and activation of caspases. CPI203 act as BH3 mimetics and help in redistribution of BIM from BFL-1-dependent complexes, and consequently could trigger the apoptotic signaling in cells exposed to CHCP treatment. Complete inhibition of cell proliferation by downregulation of MYC expression and G1 cell cycle arrest was observed after CPI203 treatment in neuroendocrine tumors^38^. Recently we showed that degradation of histone mRNA during the early S phase of the cell cycle, rather than DNA damage, is the primary cause of cancer cell death induced by CHCP^37^. How the cell cycle arrest induced by CPI203, and histone mRNA degradation induced by CAP treatment would increase the potency of cell death in combination should be determined. BAD induces apoptosis by inhibiting antiapoptotic BCL-2-family members—BCL-x, Bcl-2, thereby allowing two other pro-apoptotic proteins, BAK and BAX, to aggregate and induce release of cytochrome c, followed by caspase activation and apoptosis. BAD mRNA expression is relatively unchanged throughout the 24 h. time point suggesting that the cell death induced by CHCP treatment on breast cancer cells does not involve BAD mRNA expression. BID, BIK, and BNIP3 are pro-apoptotic members of the Bcl-2 superfamily and as such possess the ability to target intracellular membranes and contain the BH3 death domain. BID and BNIP3 mRNA expression were also downregulated throughout the 24 h time point after treatment. Whereas BIK mRNA recovery after initial downregulation suggests that BIK is involved in CHCP induced cell death along with BCL2A1. BAX mRNA expression which is regulated by the tumor suppressor P53 remained at baseline levels with brief down-regulation at 12 h time point. Both BCL2 and BAG3 mRNA were up regulated at 3 and 6 h incubation time point and down regulated by 12 h time point and again up regulated at 24 h incubation time but this differentially regulation in response to the CAP treatment were not statistically significant. BCL2 associated athanogene 1 (BAG1), a membrane protein that blocks a step in a pathway leading to apoptosis or programmed cell death. BCL2 like 10 (BCL2L10) gene has been shown to suppress cell apoptosis possibly through the prevention of cytochrome C release from the mitochondria and thus preventing caspase-3 activation. Bifunctional apoptosis regulator (BFAR) is a multidomain protein that was originally identified as an inhibitor of Bax-induced apoptosis. MCL1, BCL2 family apoptosis regulator (MCL1) an anti-apoptotic member of the B-cell lymphoma 2 (Bcl-2) family of apoptosis-regulating proteins, exemplifies several mechanisms by which a protein’s contribution to cell fate may be modified. Nucleolar protein 3 (NOL3) is an anti-apoptotic protein that has been shown to down-regulate the enzyme activities of caspase 2, caspase 8, and tumor protein p53. BAG1, BCL2L10, BFAR, MCL1, and NOL3 mRNA expressions follow a similar trend by remaining at baseline levels at 3 and 6 h. incubation time point but is slightly downregulated at 12 h and upregulated at 24 h. incubation time point. Except for CASP4 all the other Caspases that we analyzed were either unchanged or downregulated after CHCP treatment. Caspase 4 is involved in the cascade of apoptosis execution. Caspase 4 (CASP3) protein interacts with caspase-8 and caspase-9 and is activated in the apoptotic cell both by extrinsic (death ligand) and intrinsic (mitochondrial) pathways and functions as a downstream enzyme in the caspase activation cascade. How an increase in CASP4 expression either involved in cell death or survival should be determined. But CHCP treatment in combination with a pan BCL family antagonist (GA) did not show much potency, this could be because BCL2A1 overexpression has shown to be a driver of intrinsic resistance to BCL-2, BCL-XL, and MCL-1 inhibitors^39^. The only interesting exception was with BT-474 (ER^+^, PR^+^, HER2^+^), which showed the most resistance to CHCP treatment compared to all other breast cancer lines^12^, even though BT-474 does not express BCL2A1. The resistance inducing factor in this cell line remains to be determined.

Among the induction of apoptosis gene group, ABL1 that aid in intrinsic apoptotic signaling pathway via DNA damage^40^ is downregulated throughout the 24 h incubation time after CHCP treatment suggesting that enough DNA damage is not triggered after CHCP treatment at 80p 5 min condition. Similarly, the mRNA expressions of CRADD^41^ and FADD^42^, the genes involved in extrinsic apoptotic signaling pathway via death domain receptors are relatively unchanged or downregulated compared to baseline condition hint that these two are not involved in the induction of apoptosis in breast cancer cells after CHCP treatment. TP53 is a nuclear transcription factor that regulates the expression of a wide variety of genes involved in apoptosis, growth arrest, or senescence in response to genotoxic or cellular stress^43^. AKT1 is an essential serine/threonine-specific protein kinase for multiple cellular processes essential for cellular growth, metabolism, and survival was downregulated throughout the 24 h. time point after CHCP treated breast cancer cells. Among all oxidative stress genes (NOX5, GCLC, GCLM, GSR, NQO1, SQSTM1, and TXNRD1) Apolipoprotein E (APOE) showed significant upregulation after low power (80p 5 min) CHCP treatment, however, this increase was not retained at high power CHCP settings.

## Conclusion

BCL2A1 expression plays an important role in cell survival after CHCP treatment in breast cancer cells and is potentially regulated by TNF-alpha. Silencing BCL2A1 by siRNA treatment or by downregulating its expression by CPI203 treatment in combination with CHCP significantly increases the potency of the CHCP treatment. Based on our discovery a combination of CHCP and anti-BCL2A1 treatment would be beneficial and a novel therapeutic treatment option for triple negative breast cancer and other cancers.

## Materials and methods

### Cold plasma device

Canady Helios Cold Plasma Conversion System was used for performing all experiments at Jerome Canady Research Institute for Advanced Biological and Technological Sciences, Takoma Park, MD, USA. CHCP experiments were carried out as described previously^12^. Briefly, our electrosurgical device consists of the USMI SS-601 MCa high-frequency electrosurgical generator (USMI, Takoma Park, MD, USA) integrated with a USMI Canady Cold Plasma Conversion Unit and connected to a Canady Helios Cold Plasma™ Scalpel. The conversion unit has three connectors: a gas connector (to a helium tank), and an electrical connector (to the generator), and an electro-gas connector (to the scalpel). The conversion unit also features a high voltage transformer that up-converts voltage up to 4 kV, down-converts frequency to less than 300 kHz, and down-converts power less than 40 W. Additional details and schematics on plasma generation by CCPCS can be found in our previous study. The helium flow rate was set to a constant 3 L/min and the power was set to 80 and 120 P. The plasma scalpel tip was placed 1.5 cm above the surface of the cell media and remained unmoved for the duration of the treatment. The CAP treatment was performed in a laminar flow tissue culture hood, Purifier Logic + Class II, Type A2 Biosafety Cabinet (Labconco, Kansas City, MO, USA) at room temperature.

### Cell culture

Cell culture experiments were carried out as described previously^12^. Briefly, human breast cancer cell lines T-47D, SK-BR-3, and BT-474, were purchased from ATCC (Manassas, VA, USA). MCF-7, MDA-MB-231, Hs578T, and HCC1806 were generously donated by Professor Kanaan’s laboratory at Howard University. All cell lines except SK-BR-3 were cultured in Roswell Park Memorial Institute (RPMI) 1640 Medium supplemented with 10% fetal bovine serum (Sigma-Aldrich, St. Louis, MO, USA) and 1% Pen Strep (Thermo Fisher Scientific, Waltham, MA, USA) in a 37 °C and 5% CO_2_ humidified incubator (Thermo Fisher Scientific, Waltham, MA, USA). The exceptions for culture conditions include T-47D, which was additionally supplemented with 0.5 mg/mL insulin. SK-BR-3 was cultured in McCoy’s 5A medium. When cells reached approximately 80% confluence, cells were seeded at a concentration of 10^5^ cells/well into 12-well plates (USA Scientific, Ocala, FL, USA) with a 1 mL media volume per well for cell viability assays.

### Quantitative real-time RT-PCR

Total RNA was extracted from T-47D, SK-BR-3, BT-474, MCF-7, MDA-MB-231 and Hs578T cell pellets using the TRI reagent and Direct-sol MiniPrep kit (Zymo Research) with DNAse treatment according to the manufacturer's instructions. First-strand cDNA was synthesized with 1 μg of total RNA from these cells using Transcriptor First Strand cDNA Synthesis Kit (Roche Applied Science). Real-time RT-PCR reactions were performed according to the MIQE Guidelines^44^. Quantitative PCR was performed using 1 µL (diluted 1:20 using PCR grade water) of first strand cDNA under the conditions of 95 °C for 15 s, annealing at 60 °C for 60 s, extension at 72 °C for 30 s for 40 cycles, and a final extension at 72 °C for 10 min using SYBR Green Master Mix (Applied Biosciences). Primer sequences for analyzing 18S RNA were used for normalization and relative mRNA expression was calculated with 2^ − ΔΔCT^ method. Primer sequences for all the 93 genes related to induction of apoptosis, regulation of apoptosis, caspases and regulators, and responders of oxidative stress analyzed in this study are listed in Supplementary Table1.

### Western blotting

Protein lysates from cell pellets were prepared using RIPA buffer, supplemented with a complete protease inhibitor cocktail (Thermo Fisher Scientific, Waltham, MA) to prevent protein degradation. After centrifugation at 16,000 rcf for 20 min at 4 °C, protein concentrations in the supernatants were determined using the Bio-Rad Protein Assay. Twenty micrograms of protein were denatured at 95 °C for 5 min, ran on 4–20% Mini-PROTEAN^®^ TGX Stain-Free™ Protein gels (Bio-Rad), and then transferred onto Trans-Blot Turbo Mini 0.2 µm Nitrocellulose blots (Bio-Rad), according to standard protocols from Bio-Rad Laboratories (Hercules, CA)^45^. After blocking with 5% nonfat milk at 4 °C for one hour, membranes were incubated overnight with gentle agitation at 4 °C in 30 mL of blocking buffer with a mixture containing anti-BCL2A1 a polyclonal antibody from Cell Signaling (Cell Signaling Technology, Inc., Danvers, MA) or Abcam at a 1:50 dilution. After washing the blots were incubated in goat anti-rabbit HRP Ab (Bio-Rad) (1:10,000 dilution) in blocking buffer for 1 h with gentle agitation at room temperature. The blots were then incubated in Clarity western ECL substrate chemiluminescent detection reagent (Bio-Rad) for 5 min prior to automated total protein normalization on ChemiDoc MP imaging system and ImageLab software (Bio-Rad). Protein bands were analyzed by Band Analysis tools of ImageLab software version 4.1 (Bio-Rad) following standard protocol. Source data are provided as a Source Data File.

### Transfection of siRNA

Human BCL2A1 targeting MISSION^®^ esiRNA and matching scrambled control esiRNA were purchased from Sigma-Aldrich. Scrambled control esiRNA that does not target any gene was used as the negative control siRNA. MDA-MB-231 cells were transfected with siRNA and transfection reagent according to the manufacturer’s instructions. Transfection of BCL2A1 esiRNA or control esiRNA was done at 13 pmol. Briefly, cells were seeded in a 12-well-plate at a density of 1 × 10^5^ cells/well overnight in (RPMI) 1640 Medium supplemented with 10% fetal bovine serum (Sigma-Aldrich, St. Louis, MO, USA) and 1% Pen Strep (Thermo Fisher Scientific, Waltham, MA, USA) in a 37 °C and 5% CO_2_ humidified incubator. The media was replaced with antibiotics-free medium RPMI 2 min before the transfection. esiRNA was mixed with Lipofectamine RNAiMAX transfection reagent in 100 μL optimal medium at the required concentration of 13 pmol/mL and were incubated at room temperature for 30 min to form a complex and the mixture was supplemented to each well with the optimal medium. Four hours after the transfection, the transfected cells were CAP treated. After 24 h after the CAP treatment, cells viability was assessed by using the MTT assay.

### IncuCyte^®^ Cell Cycle Green/Red Reagent Stable cell line generation

One day prior to lentiviral transduction, MDA-MB-23 cells were seeded at 1 × 10^5^ cells/well on 12 well plates (Corning, NY, USA) in media supplemented with 10% fetal bovine serum (Sigma-Aldrich, St. Louis, MO, USA) and 1% Pen Strep (Thermo Fisher Scientific, Waltham, MA, USA) and incubated at 37 °C and 5% CO_2_ humidified incubator (Thermo Fisher Scientific, Waltham, MA, USA) for 24 h. Cell media was changed to fresh antibiotic-free RPMI 1640 with 10% FBS supplemented with 5 μL of 10 × virus (~ 6 MOI) IncuCyte^®^ Cell Cycle Green/Red Reagent (Sartorius, MI, USA). IncuCyte^®^ Cell Cycle Green/Red Lentivirus Reagent is a fluorescent, single cassette indicator expressing both the GFP (green fluorescent protein) and mKate2 (red fluorescent protein) to distinguish between cells in the G1 and S/G2/M cell cycle phase without altering cell function. The cells were incubated at 37 °C with 5% CO_2_ for 24 h. Cell media was changed to fresh RPMI media supplemented with 10% fetal bovine serum, 1% Pen Strep, and 5 µg/mL puromycin and incubated for 24 h. The cells with strong fluorescence and growth were selected for expansion. Selected cells were maintained in RPMI media supplemented with 10% fetal bovine serum, 1% Pen Strep, and 2.5 µg/mL puromycin for all the IncuCyte experiments.

### IncuCyte live cell imaging and cell cycle

MDA-MB-231-Cell Cycle Green/Red reagent stable cells were seeded in 12-well plates at a density of 10^5^ cells/well and treated with CAP. The treated cells were then incubated at 37 °C with 5% CO_2_ and monitored on the IncuCyte^®^ Live Cell Analysis System (Sartorius, MI, USA). The cells were scanned with phase contrast and green channels at 10 × magnification at an interval of 1 h for 3 days. Cells were scanned every hour with phase contrast, green, and red channels at 10 × magnification with Cell-By-Cell Module for MDA-MBA-231 cells. For BT-474 cells, which tend to grow in clusters, Cell by Cell Module could not properly detect the boundaries between each cell, basic scanning and analysis were used. After scanning, fluorescent objects were quantified using the IncuCyte integrated analysis software with background subtraction.

### Compounds

Gambogic acid and CPI203 were purchased from Med Chem express, USA and Sigma-Aldrich (St Louis, MO, USA) respectively.

### Gambogic acid and CPI203 treatment

Drugs were dissolved in DMSO to make stock solutions and diluted in complete growth media and subsequently added in a triplicate to the wells of 12-well plates.

### Cell viability assay

Thiazolyl blue tetrazolium bromide (MTT) assay was carried out as described previously^12^. Briefly, MTT assay was performed on the cells 24 h after plasma treatment following the manufacturer’s protocol with all MTT assay reagents purchased from Sigma-Aldrich (St. Louis, MO, USA). The absorbance of the dissolved compound was measured by BioTek Synergy HTX (Winooski, VT, USA) microplate reader at 570 nm following standard procedure.

### Statistics

Statistics analyss were performed as described previously^12^. All viability assays were repeated 3 times with at least 2 replicates each. Data was plotted by Microsoft Excel 2016 as the mean ± standard error of the mean. A student* t* test or a one-way analysis of variance (ANOVA) was used to check statistical significance where applicable. The differences were considered statistically significant for *p ≤ 0.05. A one-way multivariate analysis of variance (MANOVA) followed by a Post-Hoc test was used to check statistical significance where applicable.

## Supplementary Information

Supplementary Figures.

Supplementary Table S1.
